# Long‐Acting Technologies to Prevent HIV: Why Is the Asia‐Pacific Being Left Behind?

**DOI:** 10.1002/jia2.70112

**Published:** 2026-04-16

**Authors:** Giten Khwairakpam, Caroline Thomas, Anushiya Karunanithy, Rena Janamnuaysook, Rajkumar Nalinikanta, South Asia and South‐East Asia (SASEA) Partnership Platform

**Affiliations:** ^1^ TREAT Asia, amfAR – The Foundation for AIDS Research Bangkok Thailand; ^2^ Yayasan Peduli Hati Jakarta Indonesia; ^3^ Malaysian AIDS Council Kuala Lumpur Malaysia; ^4^ Institute of HIV Research and Innovation Bangkok Thailand; ^5^ Community Network for Empowerment Manipur India

1

Regulatory approvals of long‐acting cabotegravir (CAB LA) and lenacapavir (LEN) for HIV pre‐exposure prophylaxis (PrEP), together with their associated voluntary license agreements, have generated substantial interest from donors, national governments, licensed generic manufacturers, advocates and individuals at risk of HIV [1, 2, 3]. Civil society groups have demanded accelerated investments by donors to ensure equitable and affordable access [4, 5]. The World Health Organization (WHO) recommended CAB LA in 2022 and LEN in 2025 as PrEP options for individuals at risk [6, 7]. In 2024, the U.S. President's Emergency Plan for AIDS Relief (PEPFAR) initiated the delivery of CAB LA beyond research settings to expand access to long‐acting prevention options. In 2025, the Global Fund to fight AIDS, TB and Malaria (Global Fund) signed an agreement with Gilead Sciences, setting a goal to reach 2 million individuals with LEN over the next 3 years, with nine countries (Eswatini, Kenya, Lesotho, Mozambique, Nigeria, South Africa, Uganda, Zambia and Zimbabwe) named as early adopters [8]. The U.S. government has also announced market‐shaping support [9] to promote global production and distribution of LEN in countries with large HIV epidemics. In parallel, a new WHO prequalification process has enabled quality assurance certification in as little as 36 days [10]. Collaborations between generic companies and health agencies have resulted in a price commitment to make LEN available for procurement at $40 per person per year [11].

Many countries in Latin America, Eastern Europe and Central Asia are not included in these discussions and processes of expanding access. The Asia‐Pacific region too remains largely excluded from these global efforts to expand long‐acting PrEP options, despite facing some of the fastest‐growing epidemics worldwide. Estimates of HIV prevalence among key populations in the region remain high: 4.5% among gay men and other men who have sex with men (MSM), 7.8% among people who inject drugs (PWID), 3.1% among transgender people and 1.3% among sex workers (SW). Despite advocacy efforts and the launch of local PrEP programmes, uptake remains low, reaching fewer than 250,000 people in 2024, against a target of 8 million by 2025 [12]. HIV incidence at the national level remains a concern. For example, in Thailand, the national incidence was 2.33 per 100 person‐years (PYs) among MSM, 2.06 per 100 PYs among transgender women and 0.46 per 100 PYs among PWID, with individuals under 50 years facing a 7.30‐fold higher risk than those aged 50 years or older [13]. The Government of the Philippines has highlighted the need to address persistent rises in HIV, with reported cases increasing by 550% from 2010 to 2024 [14].

Most countries in the region rely on international funding, primarily from the Global Fund and U.S. bilateral support, to implement and support their HIV programmes. However, although the Global Fund supports a CAB LA prevention programme in Cambodia, current global donor priorities largely exclude countries in the Asia‐Pacific. This leaves them with limited support to access novel prevention tools. The majority of foreign aid for HIV prevention efforts involving oral PrEP has been concentrated in sub‐Saharan Africa, with 96% of U.S. government support alone directed towards this region [15].

The annual cost of long‐acting formulations for HIV prevention, excluding service delivery coverage, is expected to be comparable to that of the current, quality‐assured, generic oral regimen of tenofovir disoproxil fumarate/emtricitabine (TDF/FTC), which would make integration both feasible and cost‐effective [10, 11]. To enable this, national governments in the Asia‐Pacific should prioritize and increase domestic investments to sustain and scale up HIV prevention efforts to balance out, declining external donor funding. In addition, national stakeholders—including drug regulatory agencies, civil society and key population groups—should advocate for access to these products in a timely and equitable manner.

National HIV programmes in low‐ and middle‐income countries (LMICs) in the region, that are part of the voluntary licenses territory of long‐acting antiretrovirals, can leverage generic pricing and global policy developments to incorporate long‐acting HIV prevention technologies into their HIV responses. Formulating person‐centred policies, engaging key populations at high‐risk and civil society organizations, training healthcare providers, strengthening health system infrastructure to support safe and effective delivery can assist in smoothly integrating the products into existing HIV programmes. Utilization of generic products in national programmes can also encourage manufacturers to more rapidly develop and roll out generic formulations in the commercial market, potentially further driving down prices.

National drug regulators can complement efforts to expedite product registration of innovators and generic licensees. Regulatory authorities can leverage mechanisms such as the WHO Collaborative Registration Procedure or national fast‐track processes to improve efficiencies in product registration. Upper‐middle‐income countries, that are excluded from voluntary licensing agreements, such as Malaysia and China, could consider leveraging flexibilities under the Agreement on Trade‐Related Aspects of Intellectual Property Rights (TRIPS) [16] to enable generic companies manufacture for government use programmes or pursuing direct negotiations with innovator companies to ensure equitable access to the technologies and to guarantee that no one is left behind within their national contexts.

Civil society and key population groups can engage with the national Global Fund process, which is an important opportunity to introduce and scale up long‐acting HIV prevention technologies through national grant applications. Dialogue with national programme staff, Principal Recipients and Country Coordinating Mechanisms to advocate for the inclusion of long‐acting antiretrovirals for PrEP as a priority intervention in the national HIV response will be critical to keep the demand momentum up.

LMICs in the Asia‐Pacific to secure access to long‐acting antiretrovirals need simultaneous advancement of inclusive policies, efficient drug regulatory processes, upskilled healthcare personnel, active engagement of civil society and high‐risk key population groups and increased domestic funding. However, achieving control and ending HIV requires access to affordable long‐acting antiretrovirals across the global HIV response, including in Europe, Central Asia and Latin America. Ensuring that no one is left behind requires bold political will, sustained financing and meaningful community leadership. Governments and donors must prioritize equity by supporting mechanisms that ensure that these innovations reach everyone, regardless of income, geography or identity.

## Author Contributions

GK developed the concept and drafted the first version. GK, CT, AK, RJ and RN contributed ideas and reviewed the manuscript and approved the final article.

## Conflicts of Interest

RJ receives honorarium from MSD and research grants from Gilead Sciences through her organization. GK receives a grant from ViiV Healthcare through his organization. Other authors have no conflicts of interest to declare.

## Data Availability

Data sharing is not applicable to this article as no new data were created or analysed in this study.
